# Risk Factors and Correlates of School Bullying and Cyberbullying among Turkish Adolescents: Evidence from a School-Based Cross-Sectional Study

**DOI:** 10.31083/AP38859

**Published:** 2025-03-07

**Authors:** Damla Eyuboglu, Murat Eyuboglu, Didem Oktar, Seval Caliskan Pala, Zeynep Demirtas, Didem Arslantas, Alaettin Unsal

**Affiliations:** ^1^Department of Child and Adolescent Psychiatry, Eskisehir Osmangazi University Faculty of Medicine, 26140 Eskisehir, Turkey; ^2^Department of Public Health, Giresun Provincial Health Directorate, 28100 Giresun, Turkey; ^3^Department of Public Health, Eskisehir Provincial Health Directorate, 26010 Eskisehir, Turkey; ^4^Department of Public Health, Bahcelievler District Health Directorate, 34182 Istanbul, Turkey; ^5^Department of Public Health, Eskisehir Osmangazi University Faculty of Medicine, 26140 Eskisehir, Turkey

**Keywords:** cyberbullying, perpetrators, risk factors, school bullying, victims

## Abstract

**Background:**

Traditional school bullying and cyberbullying are common experiences that adversely affect the present and future mental health of adolescents. Cyberbullying has also increased during the last decade due to the growing use of the internet, mobile technological tools, and social network systems. This study aimed to investigate the risk factors of traditional school bullying and cyberbullying.

**Methods:**

The sample comprised 5491 adolescents (53.7% male and 46.3% female) from Grades 7 to 12 in 15 public schools. Participants were administered a self-report survey, including sociodemographics, school bullying, cyberbullying, and related variables. A logistic regression analysis was performed to examine the factors related to school bullying and cyberbullying involvement.

**Results:**

Boys were more prone to be perpetrators or victim-perpetrators of both school and cyberbullying. The results revealed that carrying a cutting tool, short sleep duration, using the computer and mobile phone longer, and poor academic performance were risk factors for being a perpetrator (*p* < 0.05). In addition, school bullying involvement was related to thin or overweight body perception (*p* < 0.001). Regression analysis indicated that being a victim or perpetrator of school bullying showed more significant risks for being a victim or perpetrator of cyberbullying (*p* < 0.001).

**Conclusions:**

We found common risk factors for both types of bullying and an overlap between school bullying and cyberbullying. These findings should be considered for developing new intervention programs and policies for preventing bullying in Turkey.

## Main Points

1. The study confirms that traditional bullying and cyberbullying are highly prevalent among adolescents, with significant overlap between the two forms.

2. Family dynamics, including parental separation and living arrangements, play a crucial role in determining adolescents’ vulnerability to both traditional and cyberbullying.

3. Body image perception, rather than actual physical characteristics, significantly influences the likelihood of being involved in cyberbullying, particularly as a victim.

4. Short sleep duration is associated with an increased risk of aggressive behaviors and bullying perpetration, highlighting the importance of healthy sleep patterns in adolescents.

5. Excessive use of computers and mobile devices is a strong predictor of both school bullying and cyberbullying, suggesting a need for monitoring screen time as part of prevention strategies.

## 1. Introduction

Bullying is defined as intentional and repeated aggressive behavior against less powerful individuals [1]. Recent studies performed in different countries have demonstrated that bullying and cyberbullying are common and major sources of victimization among children and adolescents and affect the well-being, physical and mental development, and academic achievement of youth [2, 3, 4, 5, 6].

Three types of bullying involvement were defined as perpetration, victimization, and both (perpetration and victimization). Traditional bullying can be physical (e.g., hitting, pushing), verbal (e.g., threatening, name-calling, teasing), sexual, or relational (e.g., spreading rumors, social excluding) [7, 8]. Cyberbullying is often described as any act of bullying performed by an individual or group using the internet or electronic devices such as mobile phones against others [9]. Many adolescents spend most of the day at school with their friends, and they continue to communicate with their friends on the Internet when they go home in the evening. What happens at school during the day continues to be discussed online in the evening and shared the next day. For this reason, cyberbullying should not be considered separately from traditional bullying [10, 11].

A meta-analysis demonstrated that the mean prevalences of traditional and cyberbullying were 35% and 15%, respectively [12]. The researchers emphasized the influential association between mental health problems and bullying involvement [13, 14]. According to current knowledge, the findings supported that depression and suicidal thoughts are associated with any bullying involvement alongside the other psychological outcomes including sleep problems, low self-esteem, relationship problems, and academic difficulties while being a perpetrator is associated with later antisocial behavior, substance use, and violence [15, 16, 17, 18, 19]. These alarming data demonstrate a crucial need for understanding the factors related to bullying involvement and mental health problems among children and adolescents.

Although previous research has extensively explored the prevalence and psychological impacts of bullying and cyberbullying, there is limited understanding of the combined role of individual, familial, and school-related factors in explaining both traditional and cyberbullying in non-Western countries. Prior studies revealed that cyberbullying was prone to increase in 11–15 years due to growing mobile phone and internet use during this period. Cyberbullying is performed by an anonymous individual or group [20]. A meta-analysis reported that cyberbullying was more related to suicidal ideation than traditional bullying [21].

Many studies have been conducted to understand traditional bullying and cyberbullying and to identify protective and risk factors. Nevertheless, at this point, it will be more understandable to interpret risk and protective factors from the framework of ecological system theory. According to the ecological systems model theory of Bronfenbrenner (1979) [22], the behaviors and experiences of young people are shaped by individual characteristics and a wide range of intertwined contextual systems, including family, school, neighborhood, and community. In this context, individuals are embedded in interconnected and layered systems, and as children develop, they are influenced not only by the phenomena in these systems but also by the interrelationship of these systems [23]. Bullying and cyberbullying are examined in the context of ecological systems theory, which investigates individual characteristics, peer and family relationships, school-related variables, and social conditions [24, 25].

Although several studies have investigated risk and protective factors related to traditional and cyberbullying victimization in Western societies, empirical research focusing on these factors in Turkish adolescents is scarce. Additionally, limited studies have utilized an integrative theoretical framework, such as the ecological systems theory, to comprehensively analyze the multiple contextual factors influencing bullying behaviors in this population. This gap in the literature highlights the need for research that considers individual, family, and school-related characteristics simultaneously. In terms of gender, males are more prone to being perpetrators [26]. On the other hand, most studies highlight that females are at greater risk of being victimized, especially in terms of cyberbullying [27, 28]. In addition, living with both parents has been observed to protect adolescents against bullying involvement. Especially children who have been victimized multiple times are usually children who live with a single parent [29]. Additionally, negative family factors such as interparental conflict and parental separation are related to involvement in bullying [17, 30].

A positive school climate and school connectedness are associated with a lower risk of bullying involvement [31]. Furthermore, previous findings supported that lower academic achievement was associated with bullying involvement [32, 33].

Previous studies reported that being obese or overweight was also associated with bullying [34]. Body weight perception has also been related to bullying, even if the weight is within the normal range [35]. The association between sleep duration and bullying was also reported in the previous studies [36, 37]. It is not surprising that short sleep duration is associated with bullying since it is known to increase aggression [37].

In light of this scientific data, it appears that bullying is related to many individual and environmental factors. Considering the limited research in Turkey and the lack of comprehensive studies incorporating traditional bullying and cyberbullying, this study aims to fill this research gap by exploring the characteristics and risk/protective factors of bullying involvement among Turkish adolescents. Understanding these factors will provide valuable insights for developing targeted intervention programs to prevent bullying and promote adolescent well-being in Turkish contexts.

The present study had two main objectives: (1) to explore characteristics of traditional and cyberbullying involvement among Turkish adolescents, and (2) to analyze the risk and protective factors for traditional and cyberbullying involvement and compare them with those of non-involved bystanders.

## 2. Material and Method

### 2.1 Participants and Procedure

The current study was carried out in Eskisehir, a province located in the Middle Anatolian Region of Turkey. According to TurkStat data, there are 111,290 people aged 10–19 years in Eskisehir [38]. Accordingly, the population size was considered 111,290. The prevalence of traditional bullying is 35% [12] with a 2% acceptable margin of error and 95% confidence interval: Accordingly, the cluster (low, middle, high socioeconomic status) sampling method was used. The design effect was accepted as “2” and the sample size was calculated as 4286.

Eskişehir is a city with a population of 871,187 and 90% of the population lives in the city center. Therefore, the study was conducted among students in schools in the city center, which were selected using the random sampling method. Schools were clustered according to their socioeconomic status as low/medium/high and 5 schools were randomly selected from each cluster. All schools implemented the standard education program prepared by the Ministry of National Education.

All students from the selected schools were invited to participate in the study (M_age_ = 14.3; Standard Deviation_age_ = 1.8). Participants were administered a self-report survey that took respondents approximately 20 min to complete during the non-teaching sessions. Surveys with missing data were not included in the study. Participation in the study was anonymous and voluntary. Participants consisted of students enrolled in Grades 6 to 12 of 15 middle and high public schools, and 5491 students were eligible for participation. Gender composition was 53.7% boys and 46.3% girls. Among the students, 11.7% were in Grade 6, 11.1% in Grade 7, 12.5% in Grade 8, 17.9% in Grade 9, 20.6% in Grade 10, 15.4% in Grade 11, and 10.8% in Grade 12.

### 2.2 Measures

#### 2.2.1 Sociodemografic Characteristics 

Demographic data included gender, age, school grade level, parental (both mother and father) education status and occupation, and monthly family income. The collection of these variables aligns with previous studies that emphasized the impact of sociodemographic factors on adolescents’ risk of involvement in bullying and cyberbullying [1, 39].

#### 2.2.2 Bullying 

Students were asked about traditional school bullying and cyberbullying victimization and perpetration in the past 12 months after defining both forms of bullying. Students responded to four questions: (1) During the past 12 months, have you ever been bullied on the school property? (2) During the past 12 months, have you ever bullied someone on the school property? (3) During the past 12 months, have you ever experienced cyberbullying (by Instagram, Facebook, text messaging, etc.)? (4) During the past 12 months, have you ever cyberbullied (by Instagram, Facebook, text messaging, etc.) someone?

These questions were adapted based on prior studies investigating school bullying and cyberbullying through self-report measures, which have been validated in various adolescent populations [40]. Self-report surveys are frequently used in bullying research due to their reliability in capturing students’ involvement in both traditional and online bullying [12].

#### 2.2.3 Family Background

Family structure was evaluated using questions about *(a) parental marital status, (b) living with mother and/or father. *Previous studies have shown that family-related variables, such as marital status and living arrangements, are important predictors of bullying and have been measured similarly in other bullying research [41, 42].

#### 2.2.4 Academic Performance

Average grade in the last semester, grade repetition, and school absenteeism were asked to measure the academic level and school connectedness. Consistent with previous research, these variables are considered significant indicators of adolescents’ school engagement and have been linked to bullying involvement [43]. Furthermore, lower academic achievement and frequent absenteeism have been identified as risk factors for both being bullied and bullying others [44].

#### 2.2.5 Additional Variables

Average mobile/smartphone or computer usage duration, doing any sports or art pursuit, sleep routine, using spectacles, carrying a cutting tool, height and weight characteristics, and perception of their bodies were also researched as additional variables.

The inclusion of these additional factors is supported by the literature, as previous studies have associated smartphone use [45], sleep disturbances [46], and body perception [47] with bullying behaviors. By incorporating these variables, this study provides a comprehensive assessment of the potential risk and protective factors associated with both traditional and cyberbullying.

### 2.3 Data Analysis

The Statistical Package for Social Sciences (SPSS) version 23.0 (IBM Corp., Armonk, NY, USA) was used to analyze the data. Categorical variables were compared using Pearson’s Chi-square test and Fisher’s exact. According to the distribution of variables, the Student’s *t*-test or Mann-Whitey U test was used to analyze continuous variables. The means, standard deviations, and percentages (%) were provided. The participants were classified as pure victims, pure perpetrators, victim perpetrators, or uninvolved groups in cyberbullying and traditional school bullying. Chi-square tests and One-Way Analysis of Variance (ANOVA) (Post hoc Tukey analysis in multiple group comparison) were performed to examine the differences between participants involved in school bullying and cyberbullying and those who did not. Logistic regression analysis was used to identify factors related to school bullying and cyberbullying. A *p*-value of less than 0.05 was interpreted as statistically significant in all tests.

## 3. Results

A total of 1855 (33.8%) participants reported being the victim of school bullying, and 1225 (22.3%) reported being the perpetrator of school bullying during the past year. In addition, a total of 942 (17.2%) participants reported being the victim of cyberbullying, and 565 (10.3%) reported being the perpetrator of cyberbullying.

Sociodemographic characteristics and related variables were analyzed by the Chi-square test. In terms of school bullying and cyberbullying, pure perpetrators and victim perpetrators were mostly males. Grade repetition and carrying a cutting tool were more common among pure perpetrators and victim-perpetrators of both bullying types. On the other hand, having separated or divorced parents was more common in both school bullying and cyberbullying involvements (victim or perpetrator). Tables 1,2 shows the comparison and distribution of variations by the subgroups.

**Table 1.** **Distribution of variables associated with traditional school bullying**.

		Total	No involvement	Pure victim	Pure perpetrator	Victim-perpetrator	*p*
			n (%)	n (%)	n (%)	n (%)	
Age							
	Early adolescence (10–14 ages)	2657 (48.4%)	1392 (44.5%)	641(56.3%)	203 (39.9%)	421 (58.8%)	< **0.001**
	Late adolescence (15–19 ages)	2834 (51.6%)	1735 (55.5%)	498 (43.7%)	306 (60.1%)	295 (41.2%)	
Gender							
	Female	2545 (46.3%)	1541 (49.3%)	585 (51.4%)	157 (30.8%)	262 (36.6%)	< **0.001**
	Male	2946 (53.7%)	1586 (50.7%)	554 (48.6%)	352 (69.2%)	454 (63.4%)	
Grade							
	6, 7 and 8	1941 (35.3%)	941(30.1%)	505 (44.3%)	155 (30.5%)	340 (47.5%)	
	9 and 10	2111 (38.4%)	1308 (41.8%)	408 (35.8%)	191 (37.5%)	204 (28.5%)	< **0.001**
	11 and 12	1439 (26.2%)	878 (28.1%)	226 (19.8%)	163 (32.0%)	172 (24%)	
Grade repetition							
	Yes	114 (2.1%)	58 (1.9%)	18 (1.6%)	14 (2.8%)	24 (3.4%)	**0.029**
	No	5377 (97.9%)	3069 (98.1%)	1121 (98.4%)	495 (97.2%)	692 (96.6%)	
Marital status							
	Married	4857 (88.5%)	2806 (89.7%)	994 (87.3%)	441 (86.6%)	616 (86%)	**0.006**
	Divorced/separated	634 (11.5%)	321 (10.3%)	145 (12.7%)	68 (13.4%)	100 (14%)	
Living with family							
	Yes	5225 (95.2%)	2979 (95.3%)	1093 (96%)	474 (93.1%)	679 (94.8%)	0.093
	No	266 (4.8%)	148 (4.7%)	46 (4%)	35 (6.9%)	37 (5.2%)	
Family income							
	High	2122 (38.6%)	1236 (39.5%)	433 (38%)	187 (36.7%)	266 (37.2%)	
	Middle	3150 (57.4%)	1782 (57%)	654 (57.4%)	304 (59.7%)	410 (57.3%)	0.125
	Low	219 (4%)	109 (3.5%)	52 (4.6%)	18 (3.5%)	40 (5.6%)	
Body image*							
	Thin	739 (13.5%)	385 (12.3%)	163 (14.3%)	84 (16.5%)	107 (14.9%)	< **0.001**
	Normal	2928 (53.3%)	1785 (57.1%)	562 (49.3 %)	270 (53%)	311 (43.4%)	
	Overweight	1824 (33.2%)	957 (30.6%)	414 (36.3%)	155 (30.5%)	298 (41.6%)	
Carrying a cutting tool in last year							
	Yes	757 (13.8%)	252 (8.1%)	128 (11.2%)	178 (35%)	199 (27.8%)	< **0.001**
	No	4734 (86.2%)	2875 (91.9%)	1011 (88.8%)	331 (65%)	517 (72.2%)	
Being a member of a school team or sport club							
	Yes	1863 (33.9%)	979 (31.3%)	383 (33.6%)	206 (40.5%)	295 (41.2%)	< **0.001**
	No	3628 (66.1%)	2148 (68.7%)	756 (66.4%)	303 (59.5%)	421 (58.8%)	
Doing an artistic activity							
	Yes	2720 (49.5%)	1528 (48.9%)	617 (54.2%)	223 (43.8%)	352 (49.2%)	**0.001**
	No	2771 (50.5%)	1599 (51.1%)	522 (45.8%)	286 (56.2%)	364 (50.8%)	
Doing a sport regularly							
	Yes	3234 (59.8%)	1803 (57.7%)	671 (58.9%)	312 (61.3%)	448 (62.6%)	0.066
	No	2257 (41.1%)	1324 (42.3%)	468 (41.1%)	197 (38.7%)	268 (37.4%)	
Daily sleep duration							
	Shorter than 8 hours	2403 (43.8%)	1336 (42.2%)	480 (42.2%)	265 (52.1%)	322 (45%)	**0.001**
	8 hours or longer	3083 (56.2%)	1788 (57.2%)	657 (57.8%)	244 (47.9%)	394 (55%)	
Mobile phone usage duration							
	Shorter than 2 hours	2935 (53.5%)	1765 (56.4%)	637 (55.9%)	209 41.1%)	324 (45.3%)	< **0.001**
	2 hours and longer	2556 (46.5%)	1362 (43.6%)	502 (44.1%)	300 (58.9%)	392 (54.7/%)	
Computer usage duration							
	Shorter than 2 hours	4530 (82.5%)	2634(84.2 %)	944 (82.9 %)	387 (76 %)	565 (78.9%)	< **0.001**
	2 hours and longer	961 (17.5%)	493 (15.8%)	195 (17.1 %)	122 (24 %)	151 (21.1 %)	
Watching TV							
	1 hour and shorter	3658 (66.6%)	2097 (67.1%)	750 (65.8%)	351 (69%)	460 (64.2%)	0.302
	Longer than 1 hour	1833 (33.4%)	1030 (32.9%)	389 (34.2%)	158 (31%)	256 (35.8%)	

The bold data are statistically significant. *, this has been assessed based on the participants’ own perceptions.

**Table 2.** **Distribution of variables associated with cyber bullying**.

		No involvement	Pure victim	Pure perpetrator	Victim-perpetrator	*p*
		n (%)	n (%)	n (%)	n (%)	
Age						
	Early adolescence (10–14 ages)	2141 (49.9%)	278 (43.6%)	110 (42.3%)	128 (42.0%)	< **0.001**
	Late adolescence (15–19 ages)	2148 (50.1%)	359 (56.4%)	150 (57.7%)	177 (58.0%)	
Gender						
	Female	1996 (46.5%)	370 (58.1%)	73 (28.1%)	106 (34.8%)	< **0.001**
	Male	2293 (53.5%)	267 (41.9%)	187 (71.9%)	199 (65.2%)	
Grade						
	6, 7 and 8	1560 (36.4%)	201 (31.6%)	88 (33.8%)	92 (30.2%)	
	9 and 10	1681 (39.2%)	242 (38%)	86 (33.1%)	102 (33.4%)	< **0.001**
	11 and 12	1048 (24.4%)	194 (30.5%)	86 (33.1%)	111 (36.4%)	
Grade repetition						
	Yes	76 (1.8%)	11 (1.7%)	10 (3.8%)	17 (5.6%)	< **0.001**
	No	4213 (98.2%)	626 (98.3%)	250 (96.2%)	288 (94.4%)	
Marital status						
	Married	3826 (89.2%)	547 (85.9%)	224 (86.2%)	260 (85.2%)	
	Divorced/separated	463 (10.8%)	90 (14.1%)	36 (13.8%)	45 (14.8%)	**0.012**
Living with family						
	Yes	4109 (95.8%)	594 (93.2%)	241 (92.7%)	281 (92.1%)	< **0.001**
	No	180 (4.2%)	43 (6.8%)	19 (7.3%)	24 (7.9%)	
Family income*						
	High	1687 (39.3%)	213 (33.4%)	115 (44.2%)	107 (35.1%)	
	Middle	2442 (56.9%)	397 (62.3%)	132 (50.8%)	179 (58.7%)	**0.005**
	Low	160 (3.7%)	27 (4.2%)	13 (5%)	19 (6.2%)	
Body image*						
	Thin	572 (13.3%)	83 (13%)	33 (12.7%)	51 (16.7%)	
	Normal	2361 (55%)	286 (44.9%)	138 (53.1%)	143 (46.9%)	< **0.001**
	Overweight	1356 (31.6%)	268 (42.1%)	89 (34.2%)	111 (36.4%)	
Carrying a cutting tool in last year						
	Yes	451 (10.5%)	96 (15.1%)	98 (37.7%)	112 (36.7%)	< **0.001**
	No	3838 (89.5%)	541 (84.9%)	162 (62.3%)	193 (63.3%)	
Being a member of a school team or sport club						
	Yes	1439 (33.6%)	212 (33.3%)	96 (36.9%)	116 (38%)	0.293
	No	2850 (66.4%)	425 (66.7%)	164 (63.1%)	189 (62%)	
Doing an artistic activity						
	Yes	2115 (49.3%)	344 (54.0%)	111 (42.7%)	150 (49.2%)	**0.018**
	No	2174 (50.7%)	293 (46.0%)	149 (57.3%)	155 (50.8%)	
Doing a sport regularly						
	Yes	2559 (59.7%)	339 (53.2%)	161 (61.9%)	175 (57.4%)	**0.013**
	No	1730 (40.3%)	298 (46.8%)	99 (38.1%)	130 (42.6%)	
Daily sleep duration						
	Shorter than 8 hours	1799 (42.0%)	314 (49.3%)	142 (54.6%)	148 (48.5%)	< **0.001**
	8 hours or longer	2485 (58.0%)	323 (50.7%)	118 (45.4%)	157 (51.5%)	
Mobile phone usage duration						
	Shorter than 2 hours	2431 (56.7%)	286 (44.9%)	109 (41.9%)	109 (35.7%)	< **0.001**
	2 hours and longer	1858 (43.3%)	351 (55.1%)	151 (58.1%)	196 (64.3%)	
Computer usage duration						
	Shorter than 2 hours	3598 (83.9%)	539 (84.6 %)	182 (70 %)	211 (69.2%)	< **0.001**
	2 hours and longer	691 (16.1%)	98 (15.4 %)	78 (30 %)	94 (30.8 %)	
Watching TV						
	1 hour and shorter	2806 (65.4%)	458 (71.9%)	180 (69.2%)	214 (70.2%)	**0.004**
	Longer than 1 hour	1483 (34.6%)	179 (28.1%)	80 (30.8%)	91 (29.8)	

The bold data are statistically significant. *, this has been assessed based on the participants’ own perceptions.

In this study, pure perpetrators of school bullying were heavier and taller than the others. Additionally, pure perpetrators of school bullying and pure perpetrators and victim perpetrators of cyberbullying had poorer academic performance and more school absenteeism (Table 3).

**Table 3.** **Distribution of physical characteristics, academic performance, and school absenteeism by subgroups of both bullying types**.

	Traditional school bullying						Cyberbullying					
	No involvement (NI) (n = 3127)	Pure victim (PV) (n = 1139)	Pure perpetrator (PP) (n = 509)	Victim-perpetrator (VP) (n = 716)			No involvement (NI) (n = 4289)	Pure victim (PV) (n = 637)	Pure perpetrator (PP) (n = 260)	Victim-perpetrator (VP) (n = 305)		
	Mean (SD)	Mean (SD)	Mean (SD)	Mean (SD)	*p*	Post Hoc*	Mean (SD)	Mean (SD)	Mean (SD)	Mean (SD)	*p*	Post Hoc*
Height (cm)	165.6 (10.8)	163.4 (10.6)	168.4 (10.6)	165.2 (11.1)	<0.001	PP > NI = VP > PV	165 (11.1)	165.3 (9.3)	168.2 (10.5)	168.5 (11.3)	<0.001	PP = VP > PV = NI
Weight (kg)	56.1 (13.7)	54.5 (13.7)	58.9 (13.1)	56.2 (14.3)	<0.001	PP > NI = VP > PV	55.7 (13.8)	56.1 (12.8)	59.1 (14.1)	58.6 (13.8)	<0.001	PP = VP > PV = NI
Academic performance	82.5 (12.2)	84.1 (11.2)	79.1 (12.7)	81.3 (12.6)	<0.001	PV > NI = VP > PP	82.7 (12.1)	82.4 (11.9)	79.5 (12.9)	79.6 (12.8)	<0.001	NI = PV > VP = PP
School absenteeism (day)	5.3 (6.5)	5 (6.3)	7.7 (9.3)	6.4 (7.8)	<0.001	PP > VP > NI = PV	5.2 (6.7)	6.1 (6.7)	8.4 (9.1)	7.5 (8.0)	<0.001	PP = VP > PV = NI

One way Analysis of Variance (ANOVA) * Tukey. SD, Standard deviation.

According to the logistic regression analysis performed with those statistically significant in bivariate analysis, grade, parental marital status, grade repetition, being a member of a school team or sports club, average daily sleep duration, carrying cutting tools, perception of the weight, and mobile phone and computer usage duration were associated with being a perpetrator (Table 4).

**Table 4.** **The risk factors of being perpetrator: Logistic Regression Model**.

		Traditional school bullying			Cyberbullying		
		OR	95% CI	*p*	OR	95% CI	*p*
			lower-upper			lower-upper	
Age							
	Early adolescence:10–14 years	1.105	0.871–1.402	0.410	1		
	Late adolescence: 15–19 years	1			0.949	0.686–1.312	0.750
Gender							
	Male	1.615	1.391–1.875	< **0.001**	1.443	1.182–1.763	< **0.001**
Grade							
	6–7–8th grades	1.933	1.540–2.426	< **0.001**	1.272	0.928–1.744	0.135
	11–12th grades	1.186	0.976–1.441	0.085	1.464	1.142–1.876	**0.003**
Marital status							
	Seperated/divorced	1.229	1.005–1.504	**0.045**	1.197	0.916–1.563	0.187
Grade repetition in the last year							
	Yes	1.290	0.842–1.975	0.242	2.192	1.360–3.534	**0.001**
Doing a sport regularly							
	Yes	0.981	0.841–1.145	0.810	0.906	0.741–1.110	0.341
Being a member of a school team or sport club							
	Yes	1.325	1.144–1.536	< **0.001**	0.838	0.689–1.020	0.078
Doing any artistic activity							
	No	1.105	0.961–1.272	0.162	1.010	0.839–1.215	0.917
Average daily sleep duration							
	Shorter than 8 hours	1.213	1.049–1.403	**0.009**	1.225	1.012–1.482	**0.037**
Cutting tool carriage in the last year							
	Yes	4.003	3.377–4.744	< **0.001**	3.763	3.071–4.611	< **0.001**
Perception of weight							
	Overweight and obese	1.355	1.167–1.574	< **0.001**	1.087	0.835–1.417	0.535
	Thin	1.295	1.059–1.583	**0.012**	1.088	0.893–1.325	0.402
Mobile phone usage duration							
	More than 2 hours	1.781	1.543–2.055	< **0.001**	1.922	1.588–2.325	< **0.001**
Computer usage duration							
	2 hours and longer	1.223	1.033–1.449	**0.02**	1.974	1.605–2.427	< **0.001**
	Constant	0.738		<0.001	0.635		<0.001

Logistic regression analyses, *p* < 0.001 in Omnibus test of model coefficients. OR, Odds ratio; CI, Confidence interval; 1, Reference. The bold data are statistically significant.

Furthermore, according to the logistic regression analysis performed with those statistically significant in bivariate analysis, gender, grade, family income, perception of body weight, doing an artistic activity, and carrying a cutting tool were associated with being a victim (Table 5).

**Table 5.** **The risk factors of being a victim: Logistic Regression Model**.

		Traditional school bullying			Cyberbullying		
		OR	95% CI	*p*	OR	95% CI	*p*
			lower-upper			lower-upper	
Age							
	Early adolescence: 10–14 years	1.117	0.919–1.357	0.268	1		
	Late adolescence: 15–19 years	1			1.022	0.800–1.305	0.861
Gender							
	Female	1.083	0.958–1.225	0.204	1.283	1.100–1.496	**0.001**
Grade							
	6–7–8th grades	1.984	1.544–2.550	< **0.001**	0.923	0.768–1.111	0.399
	9–10th grades	1.070	0.905–1.265	0.430	1.285	1.084–1.522	**0.004**
Marital status							
	Seperated/divorced	1.189	0.997–1.418	0.054	1.208	0.976–1.494	0.082
Living with family							
	No	0.951	0.722–1.253	0.722	1.429	1.061–1.926	**0.019**
Family income							
	Middle income	1.186	1.049–1.341	**0.006**	1.224	1.049–1.429	**0.010**
	Low income	1.623	1.210–2.178	**0.001**	1.297	0.900–1.867	0.163
Grade repetition in the last year							
	Yes	1.030	0.695–1.527	0.881	1.573	1.010–2.450	**0.045**
Being a member of a school team or sport club							
	No	1.087	0.962–1.229	0.181	1.175	1.002–1.379	**0.047**
Doing a sport regularly							
	No	0.950	0.844–1.070	0.401	1.181	1.019–1.369	**0.028**
Doing any artistic activity							
	Yes	1.055	0.938–1.186	0.375	1.252	1.077–1.454	**0.003**
Average daily sleep duration							
	Shorter than 8 hours	1.160	1.024–1.313	**0.020**	1.091	0.936–1.271	0.265
Cutting tool carriage in the last year							
	Yes	1.742	1.480–2.049	< **0.001**	1.974	1.634–2.385	< **0.001**
Perception of weight							
	Overweight and obese	1.590	1.381–1.831	< **0.001**	1.400	1.196–1.638	< **0.001**
	Thin	1.359	1.132–1.631	< **0.001**	1.210	0.972–1.506	0.087
Mobile phone usage duration							
	More than 2 hours	1.335	1.182–1.509	< **0.001**	1.648	1.415–1.920	< **0.001**
Computer usage duration							
	2 hours and longer	1.026	0.882–1.194	0.737	1.134	0.944–1.361	0.179
	Constant	0.473		<0.001	0.213		<0.001

Logistic regression analyses, *p* < 0.001 in Omnibus test of model coefficients. OR, Odds ratio; CI, Confidence interval; 1, Reference. The bold data are statistically significant.

Furthermore, 65% of school bullying perpetrators have reported themselves as cyberbullying perpetrators as well. Victims of school bullying (odds ratio (OR) = 1.491, 1.218–1.826, *p* < 0.001) and perpetrators of school bullying (OR = 6.406, 5.210–7.876, *p* < 0.001) were more likely to become perpetrators of cyberbullying.

Lastly, 61.1% of cyber victims have reported themselves as a victim of school bullying as well. Being a victim of school bullying (OR = 3.533, 3.019–4.133, *p* < 0.001) and being a perpetrator of school bullying (OR = 1.941, 1.637–2.300, *p* < 0.001) showed a higher risk of cyber victims.

## 4. Discussion

School bullying and cyberbullying are common phenomena raising many concerns for adolescents, parents, and teachers. To date, research has mostly focused on victimization or perpetration. In this study, we aimed to investigate the risk factors related to both sides of bullying, victimization, and perpetration. Consistent with the previous studies, the results showed that the prevalence of being a victim of school bullying was 33.8%, and being a victim of cyberbullying was 17.2%. However, the prevalence of bullying varies from country to country, the percentage of school bullying and cyberbullying in this study were similar to a meta-analysis performed by Modecki *et al*. [12], which found 35% and 15%, respectively.

Considering differences by gender, the risk of being exposed to cyberbullying is higher in females than in males, which is consistent with previous research [39, 48, 49]. The reasons for this result are that females are greatly likely to be exposed to bullying from both genders [49], and that the violence against women is common in Turkey; therefore, it may have been culturally internalized by both genders. Additionally, consistent with the previous studies, our study showed that males were more tend to be perpetrators of two types of bullying [50, 51, 52]. Literature contains several studies that reported mixed findings regarding gender differences. These results may have been conditioned by the methodological differences of the studies (sampling, age groups, questionnaires, types of bullying researched) and cultural diversities of countries.

Our study supported that although traditional school bullying involvement (perpetration, victimization, or both) was more common in middle schools, it tended to decrease in high schools. Research has shown that younger children are more prone to being involved in traditional bullying [49, 53]. Consistent with the previous studies, this study showed that cyberbullying involvement rates increased in high schools and there were greater odds of being cyber perpetrators in Grades 11 and 12 compared to Grades 9 and 10, while being a victim of cyberbullying rates were constant in all grades [52, 54]. Robson and Witenberg (2013) [52] reported that older students were more prone to being cyberbullies compared to younger students. The reasons for increasing cyberbullying involvement in adolescence might be increasing social networking services and internet usage duration, improving social skills, and growing demand for social relationships.

The results of this study align with the ecological model, emphasizing that bullying involvement is influenced by a complex interplay of individual characteristics (e.g., gender, academic performance, body perception) and contextual factors (e.g., family structure, school environment). This framework reveals a comprehensive understanding of bullying behaviors as multidimensional phenomena shaped not only by personal attributes but also by the broader ecological systems in which adolescents are embedded. Specifically, our findings suggest that adolescents’ engagement in bullying is modulated by factors operating at multiple levels, including family context, peer relationships, and school dynamics, supporting the assumptions of Bronfenbrenner’s ecological systems theory.

This study has contributed several new insights that were not emphasized in previous research. Firstly, our findings indicated that both being a victim and a perpetrator of traditional school bullying were strongly associated with increased involvement in cyberbullying, suggesting that these two forms of bullying might not be entirely distinct phenomena but rather interconnected behaviors that may overlap significantly in the adolescent population. Secondly, the study revealed a unique pattern in family-related risk factors, where students from separated or divorced families were at a higher risk of traditional bullying perpetration; yet there was no such association with cyberbullying involvement. This finding differs from some previous Western studies, indicating that cultural factors might play a significant role in the dynamics of family context and bullying behaviors.

Furthermore, the ecological model underscores the importance of considering both proximal and distal influences when examining bullying behaviors. For instance, while individual traits such as body perception play a critical role in determining vulnerability to victimization, contextual factors like family support and school climate also serve as crucial moderators of these associations. Our findings suggest that future research should adopt a more nuanced approach that simultaneously assesses individual, familial, and school-related variables to fully capture the multifaceted nature of bullying involvement.

### 4.1 Family Context

This study indicated that having divorced or separated parents and not living with the family were more common characteristics in the bullying involvement group. For traditional school bullying perpetration, having separated parents was a risk factor. Nevertheless, there was no association between being a cyber perpetrator and parental marital status. Early studies have reported that family conflict showed a predictive association between bullying and aggression [17, 30]. Additionally, our study supported that living with the family was a protective factor for being a victim of cyberbullying. This result is in line with the other studies in which the rates of victimization have been lesser in intact families [51, 52]. Consistent with the previous studies, adolescents with middle and low family income were more likely to be victims of two bullying types [49]. On the other hand, for students from low-income families, there was no link between cyber victimization and family income. This might be due to not having personal smartphones or computers.

A novel finding in this study was that while living in an intact family was a protective factor against cyberbullying victimization, it did not have a significant protective effect on traditional bullying involvement. This contrast highlights the need to consider different family structures and dynamics within the ecological framework when designing interventions for different types of bullying.

### 4.2 School Context

Our results demonstrated that school absenteeism and low academic performance rates were higher in pure perpetrators. These results confirmed the previous studies reporting that school climate, academic performance, school connectedness, and skipping lessons without permission were related variables to bullying [31, 33]. Although pure victims of school bullying had better academic performance, there was no significant difference between cyberbullying victims and uninvolved. Therefore, students with better academic performance might be a target for traditional school bullying. We found grade repetition as a risk factor for cyberbullying, as both perpetrators and victims. In our country, grade repetition is mostly due to academic failure or school absenteeism.

A novel contribution of our study was identifying distinct academic patterns associated with traditional and cyberbullying. While high academic performance was a risk factor for being a victim of traditional bullying, it was not significantly linked to cyberbullying involvement. This suggests that better-achieving students might be targeted in face-to-face school environments yet not necessarily in online settings.

Future research should consider the broader educational context, including peer dynamics and teacher-student interactions, to better understand how school environments shape different types of bullying behaviors within the ecological model. This will provide a more comprehensive perspective on how to structure school-based intervention programs.

### 4.3 Individual Context

The participants were compared by their physical features, and school bullying perpetrators were heavier and taller than the victims. Hence, pure perpetrators were more bodied than pure victims. Olweus (2013) [1] emphasized the power imbalance as a characteristic of school bullying, so our findings were consistent with this description. In our study, we also asked the participants about their body perception, and thin and overweight body perceptions were related to school bullying involvement. Ganapathy *et al*. (2019) [55] also reported that being obese or the perception of being obese was associated with bullying. Cyber victims were more prone to perceive their bodies as overweight. Consistently, Merrill and Hanson (2016) [49] described that students who had weight problems were more vulnerable to bullying. For cyberbullying, there was no association between perpetration and body shape. It might be due to remaining anonymous for cyber perpetrators. Although average weight and height rates were not different between the cyber victims and the uninvolved group, cyber victims more frequently reported themselves as overweight or obese. In the previous studies, the victims of cyberbullying had poor body image perception and dissatisfaction with appearance [51, 56, 57]. Our study highlighted a novel finding that adolescents’ perceptions of their bodies, rather than actual physical characteristics, were more significantly related to cyberbullying victimization, which was not the case for traditional bullying. This suggests that self-perception and psychological vulnerability might be more critical factors in understanding cyber victimization.

Our results indicating an association between being a member of a school team or a sports club and being a school bullying perpetrator is in line with previous studies, which reported a relation between violence due to competition and team sports [58]. Another reason for this relationship may be that being a member of a peer group is a sign of power during adolescence. Interestingly, while there was no relation between doing any sport regularly and any bullying involvement, students who did not do any sport regularly were more likely to become victims of cyberbullying. Physical activity or doing any sport regularly is a part of a healthy lifestyle. Some researchers emphasized that victimization was related to unhealthy and sedentary lifestyles and spending more time on computers or smartphones [49]. It is hard to interpret this association according to our findings, ultimately. Yet we think it might be due to dissatisfaction with body image and life, low self-esteem, and not taking enough care of themselves.

In the present study, we also investigated artistic activities (painting, drawing, playing a musical instrument, etc.). Artistic activities were found to be more common among victims of two bullying types than perpetrators, and doing these activities was found to be a risk factor for cyber victimization. We have not come across any pre-existing research investigating this relationship in the literature. But, Woods and Hampson (2010) [59] reported that artistic occupations were more likely for women than men. Therefore, higher rates of cyber victims’ artistic activities might be due to the high rate of females. And, the students involved in artistic activities might become easily a target for bullying. Furthermore, we think those adolescents might also have emotional-sensitive-withdrawn personality traits. Besides, victims may engage in artistic activities to deal with the difficult emotions because of bullying.

The literature contains many studies emphasizing the association between both types of bullying and their negative mental health consequences [4]. Recent studies reported a link between bullying and sleep problems [60, 61]. Hunter *et al*. (2014) [62] reported high insomnia rates among adolescents who were victims, bullies, and bully-victims. Consistent with the previous studies, our findings indicated that short sleep duration is common among perpetrators of both bullying types. However, sleep duration shorter than 8 hours was a risk factor for being a perpetrator. Short sleep duration may be related to an emotional problem caused by bullying, or it may be related to other mental disorders that make adolescents more prone to bullying involvement [60, 61]. Additionally, short sleep duration is known to make individuals more prone to aggression [37].

In our current study, the duration of the use of computers and mobile phones was longer among adolescents who were traditional and cyberbullying perpetrators. Moreover, we found that two hours and longer use of computers and mobile phones was a risk factor for being perpetrators of both types of bullying, and cyber victims. Previous studies found that cyberbullying was related to the amount of time spent on the internet or social media [9, 63, 64]. Our findings supported that school bullying perpetrators spent more time on computers and mobile phones as well. We did not investigate the mobile phone or computer usage purposes; however, one of the reasons might be that school bullying perpetrators used these tools for video games, watching videos, etc., and not only social media or connecting with others. Besides, it might also be the case that cyberbullies and school bullies are almost the same students. Supporting this, our findings demonstrated that sixty-five percent of school bullies were also cyberbullies, and according to regression analysis, being a perpetrator of school bullying increased the risk of being a cyberbully six times.

The results of our study showed that being a victim of traditional school bullying increased the risk of cyberbullying victimization three or four times. Other studies have pronounced the different overlapping rates between cyber and school bullying [64]. Previous studies carried out over 80% of cyber victims and cyberbullies were also school victims and bullies [12, 65].

Finally, in our study, we investigated the cutting tool carrying and its relationship with bullying. Our study results showed that pure perpetrators and victim-perpetrators of both types of bullying carried much more cutting tools. However, victims of both types of bullying also carried more cutting tools than uninvolved students. According to our study, carrying cutting tools increased the risk of being a perpetrator four times and the risk of being a victim two times for both types of bullying. These risks might be bidirectional. Consistent with our findings, recent meta-analyses revealed that adolescents involved in bullying as a victim, bully, or bully-victim had higher odds of carrying a weapon than uninvolved adolescents [66, 67]. Cutting tools may have different functions depending on their role in bullying. Perpetrators may use them to intimidate others [68]. Moreover, victims may protect themselves by carrying a cutting tool, and victim-perpetrators may likely carry cutting tools for both purposes [67].

This current study has several limitations. The first one is that the findings of the current study were based on responses to a self-report survey. Therefore, the data we collected are vulnerable to recall bias. The second limitation is the cross-sectional design of the study which was carried out at a certain period. This study design is not convenient for making inferences about the causality of related data, which may have bidirectional associations. Longitudinal studies are needed to find out critical factors for bullying. The other limitation is that students were from public schools; therefore, we lack data from private schools. The last limitation of our study is that we did not investigate the types of bullying (physical, verbal, social, etc.).

In conclusion, the focus of this present study was to investigate the risk factors of traditional school bullying and cyberbullying. We compared the variables according to pure victims, pure perpetrators, victim perpetrators, and uninvolved groups. Subsequently, we tried to identify risk factors for victims and perpetrators based on logistic regression analysis. Since some variables mentioned above were common in both bullying types and both roles, we assumed that it was hard to separate the types and roles of bullying each other. Supporting this, our study demonstrated high rates of overlapping and being a risk factor for each other.

In addition, our study indicated high percentages of traditional school bullying and cyberbullying in the study sample, which might represent our country. The intervention programs for preventing bullying should be developed according to our culture and made available for students, parents, educators, clinicians, and policymakers. Besides, more attention should be paid to school bullying to prevent cyberbullying, which may continue until early adulthood and result in cybercrime.

Future research should focus on longitudinal studies to explore the causal relationships between risk factors and bullying involvement to clarify the temporal dynamics of these associations. In particular, studies monitoring the developmental trajectory of bullying behaviors across different school levels (elementary to high school) are recommended to determine the transition patterns between traditional and cyberbullying. Moreover, it is essential to investigate the role of cultural and family factors in depth to understand how cultural norms, family structure, and parental practices influence adolescents’ risk of being involved in both types of bullying. Future research could also benefit from examining specific intervention strategies that target identified risk groups, such as high-achieving students for traditional bullying or adolescents with negative body perceptions of cyberbullying.

Given the significant overlap between school bullying and cyberbullying observed in this study, future studies should adopt an integrative approach that considers both forms of bullying simultaneously. This will help to determine whether prevention strategies developed for school bullying are also effective in reducing cyberbullying rates or whether unique strategies are required for each type. Finally, researchers should investigate the potential role of digital literacy and online behavior education as preventive measures for cyberbullying, particularly in settings where internet usage rapidly increases among adolescents.

## 5. Conclusions

This study highlights the intricate relationships between traditional school bullying and cyberbullying among Turkish adolescents, emphasizing shared risk factors and overlapping involvement in both forms. The findings underscore the need for culturally tailored intervention programs that address individual, family, and school-related risk factors. Future research should focus on longitudinal studies to explore causal relationships and develop targeted strategies for prevention. Addressing these issues comprehensively will contribute to promoting adolescent well-being and reducing the prevalence of bullying in Turkey.

## Availability of Data and Materials

The corresponding author will provide the data that underpin the study’s conclusions with a reasonable application.
